# UTILIZING EFFECTIVE MENTORING STRATEGIES FOR RESEARCH SUCCESS: NYU SUMMER SCHOLARS PROGRAM

**DOI:** 10.1093/geroni/igz038.2782

**Published:** 2019-11-08

**Authors:** Meredeth Rowe, Tracey Yap, Bei Wu

**Affiliations:** 1 University of South Florida, Tampa, Florida, United States; 2 Duke University, Durham, North Carolina, United States; 3 New York University, New York, New York, United States

## Abstract

Since 1998 the Hartford Institute for Geriatric Nursing has offered a summer program designed to accelerate the development of research projects and trajectories for young scientists. This interprofessional program brings faculty and scholars together from across the globe representing all healthcare disciplines. In the week long program, scholars benefit from expert presentations and daily mentoring sessions with experts in gerontological research. In this symposium, scholars will discuss strategies they have used to successfully move from ideas for research, to development of proposals, conduct of research projects and planning career trajectories. We will present differing mentoring strategies and available mentoring opportunities for gerontological-focused scientists. Each scholar will present their work with topics including optimal medication management to sustain home placement: use of adaptive dance to improve mood and function in persons with Alzheimer’s disease; facilitating positive attitudes toward older adults in nursing students; the use of mindfulness to reduce symptoms of urinary incontinence; and, a novel intervention to reduce pressure ulcers in the nursing home.

